# The potential of nano graphene oxide and chlorhexidine composite membranes for use as a surface layer in functionally graded membranes for periodontal lesions

**DOI:** 10.1007/s10856-023-06767-7

**Published:** 2023-12-16

**Authors:** Syed Saad Bin Qasim, Jasim Ahmed, Maribasappa Karched, Adel Al-Asfour

**Affiliations:** 1 Department of Bioclinical Sciences, College of Dentistry, Kuwait University, Kuwait, Kuwait; 2 Environment and Life Sciences Research Center, Kuwait Institute for Scientific Research, Safat, Kuwait; 3 Department of Biological Sciences, College of Dentistry, Kuwait University, Kuwait, Kuwait; 4 Department of Surgical Sciences, College of Dentistry, Kuwait University, Kuwait, Kuwait

## Abstract

Membranes have been used for treating periodontal defects and play a crucial role in guided bone regeneration applications. Nano graphene oxide have been exploited in tissue engineering due to its biomechanical properties. Its composite formulations with hydroxyapatite and chitosan with controlled degradation could aid in becoming part of a surface layer in a functionally graded membrane. The aim of the study was to synthesize chitosan and composite formulations of nano graphene oxide, hydroxyapatite and chlorhexidine digluconate using solvent casting technique and to characterize the physiochemical, mechanical, water vapor transmission rate (barrier), degradation and antimicrobial potential of the membranes. Altogether four different membranes were prepared (CH, CCG, 3511 and 3322). Results revealed the chemical interactions of hydroxyapatite, chitosan and nanographene oxide due to inter and intra molecular hydrogen bonding. The tensile strength of 3322 (33.72 ± 6.3 MPa) and 3511 (32.06 ± 5.4 MPa) was higher than CH (27.46 ± 9.6 MPa). CCG showed the lowest water vapor transmission rate (0.23 ± 0.01 g/h.m^2^) but the highest weight loss at day 14 (76.6 %). 3511 showed a higher drug release after 72 h (55.6 %) Significant biofilm growth inhibition was observed for all membranes. 3511 showed complete inhibition against *A. actinomycetemcomitans*. Detailed characterization of the synthesized membranes revealed that 3511 composite membrane proved to be a promising candidate for use as a surface layer of membranes for guided bone regeneration of periodontal lesions.

**Graphical Abstract:**

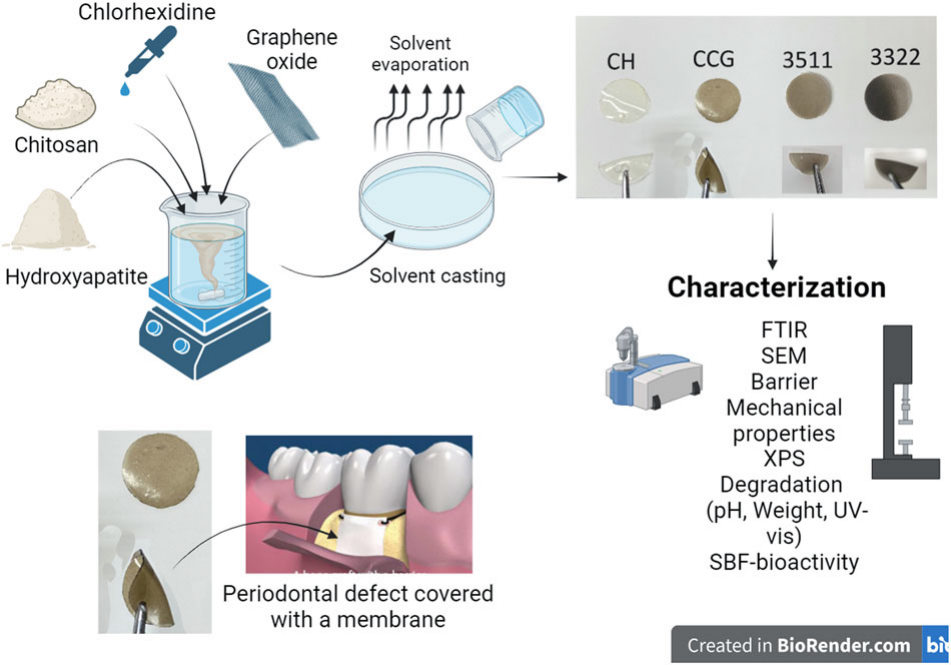

## Introduction

Contemporary therapeutic approaches within the realm of periodontology are centered on both non-surgical and surgical interventions for managing periodontal disease. While these modalities yield comparable long-term treatment outcomes, surgical intervention engenders more pronounced reductions in probing depth and gains in clinical attachment level within sites initially characterized by deep periodontal pockets [1]. The application of guided tissue regeneration (GTR) membranes in surgical periodontics has exhibited encouraging outcomes, owing to its foundation on robust biological principles. The rationale underlying this approach involves the placement of a physical barrier to selectively facilitate cellular proliferation by impeding the ingress of epithelial cells, gingival connective tissue, and other cellular elements that contribute to fibrotic responses [2]. Membranes used in surgical periodontics come in the form of resorbable and non-resorbable options. The concept of functionally graded membrane (FGM), that is biologically active and spatially designed was proposed as the next generation of biomimetic membranes by Bottino et al. [3, 4]. Since then, researchers have proposed different fabrication techniques of such templates using electrospinning, freeze gelation / drying [5] and solvent casting [2]. With the advent of novel nanomaterials like nano graphene oxide (GO) [6], boron nitride [7] and silicon nitride, different composite formulations of either natural and synthetic polymers, bioceramics and biomolecules have been used to synthesize these membranes. Investigators across the globe are still in search of the ideal surface or core layer of FGM. Nevertheless, each layer needs to be tailored individually to trigger a rapid initiation of guided bone regeneration (GBR) [8].

Although the use of such membranes has shown substantial results with respect to regeneration of bone and tooth supporting structures, bacterial contamination is the most significant factor jeopardizing treatment outcomes. Moreover, it has also been reported that the membranes become contaminated at the initial 3 min (mins) of GTR procedure with different Gram-positive and periodontal pathogens [9]. Chitosan (CH) which is derived from the second most abundantly available natural amino polysaccharide, chitin, has shown promising results due to its biophysical and chemical properties in tissue engineering applications. Its inherent antimicrobial nature and ability to degrade have been exploited by investigators to synthesize composite formulations in the form of membranes, fibers and porous templates with other nano-biomaterials like hydroxyapatite (HA) and collagen [10–12]. Previous studies have investigated the potential of solvent casting as a convenient method for membrane fabrication whilst using CH and HA composites. They proposed that the future of synthesizing such membranes that could act as a surface layer in a FGM lies in harnessing the potential of novel nanomaterials and composite formulation with drugs to combat the post-surgical infections. Recently, a revolutionary nanomaterial that has shown great potential in tissue regeneration, drug delivery and other biomedical applications is GO [13]. GO has demonstrated the capability to induce osteogenic differentiation of stem cells and incite an antimicrobial response *via* diverse mechanistic pathways. Its large surface area facilitates the adsorption of aromatic compounds, hydrogen bonding, and assorted interaction [14]. Similarly, chlorhexidine digluconate (CHX) has a long standing history of use in GBR procedures. It has also been used in composite formulations due to its broad range of antibacterial spectrum [9].

Therefore, the aim of the current study was to synthesize and comprehensively characterize CH based membranes with GO, HA and CHX that could potentially serve as a surface layer in a FGM to evaluate their biophysical, chemical, mechanical and antimicrobial potential using Scanning electron microscopy (SEM), Fourier transform infrared spectroscopy (FTIR), X-ray photoelectron spectroscopy (XPS), barrier properties, swelling and degradation profile using pH analysis, weight profile and Ultraviolet-visible (UV-vis) spectroscopy. The objective was to characterize the physiochemical, mechanical, biological and antimicrobial properties of the synthesized membranes. The antimicrobial potential was analyzed using bacterial CFU from microbial cultures and cell numbers from quantitative polymerase chain reaction (qPCR) was also conducted.

## Materials and methods

Medium molecular weight CH (≥75% deacetylation, 190,000 to 310,000 Da; Sigma Aldrich, Burlington, MA, USA), Ultra Highly Concentrated Single-Layer Graphene Oxide (Color: brown; concentration: 6.2 g/L; and flake size: 0.5 to 5 µm) (Graphene Supermarket^®^, Graphene Laboratories Inc., Ronkonkoma, NY,USA); NanoXIM-Care Paste (nano-hydroxyapatite water-based paste; size of HA nanoparticles: <50 nm; surface area: ≥80% m^2^/g) (Fluidinova, Portugal). The HA paste was oven-dried at 60 °C for 72 h, followed by heating at 500 °C for 24 h in a muffle furnace. The obtained powder was thereafter used for synthesizing composite membranes. All other reagents and chemicals (e.g., acetic acid, sodium hydroxide, ethanol, and CHX were procured from Sigma Aldrich (Burlington, MA, USA).

### Synthesis of membranes

The CH-based membranes were prepared by solvent casting technique [2]. The neat CH membranes were synthesized by dissolving the required amount of the polymer in 0.2 M acetic acid at a concentration of 2% (w/v), followed by the addition of 0.5 mL / g of glycerol. The CH / glycerol blend solution was stirred at 40 °C for 4 h, followed by centrifugation at 4000 rpm for 20 min (minutes), and finally, the solutions were cast in 8 cm-diameter plastic petri dishes. These solutions were left to air dry at ambient temperature for 24 h (hr). The dried membrane was carefully peeled off and stored in sealed bags inside a desiccator containing saturated silica.

For the synthesis of composite membranes, pre-selected ratios of GO, HA, and CHX were added to CH solutions. CH/CHX/GO were used at a ratio of 50:25:25 (CCG). HA based membranes were synthesized at a ratio of CH/HA/CHX/GO: 30:50:10:10 (CHCG 3511) and 30:30:20:20 (CHCG 3322). GO, CHX, and HA were added in their respective amounts prior to the addition of acetic acid. These membranes were casted following the same steps as mentioned earlier for CH membranes. The prepared membranes were neutralized with a 1 M NaOH and 100% ethanol mixture (1:1) for an hr, followed by washing twice with phosphate buffered saline (PBS) for 15 min.

### Scanning electron microscopy

Membrane specimens were mounted on aluminum stubs with carbon tape and sputter coated with gold (Structure Probe, West Chester, PA). Images were acquired using SEM (JEOL, JSM- IT200 InTouchScope™ Tokyo, Japan) at two different magnifications of 100 × and 330×. The SEM images were captured at an accelerating voltage of 10 kV. The raw images were transferred to ImageJ software (National Institutes of Health, Bethesda, Maryland, USA) for rescaling.

### Fourier transform infrared (FTIR) spectroscopy

FTIR analysis was performed in Attenuated total reflectance mode (ATR) on the Bruker Tensor 27 System (Bruker Optics Inc., Ettlingen, Germany) by placing the sample in contact with the diamond crystal. 16 scans per specimen were recorded at a resolution of 8 cm^−1^ from a 500 to 4000 cm^−1^ wavenumber using the instrumental software. The acquired data were corrected by the ATR correction of the instrumental software (OMNIC^™^; Version 8.2.0, Thermo Scientific, Waltham, MA, USA).

### X-ray photoelectron microscopy (XPS)

ESCALAB™ QXi X-ray Photoelectron Spectrometer (XPS) (Thermo Scientific, Waltham, MA, USA), equipped with a monochromator Al Kα X-ray irradiation source of power 1486.5 eV and charge compensation flood gun, was used in the membrane surface investigation. Prior to the XPS measurements, the instrument was calibrated with Au, Cu, and Ag metals. Each specimen was fixed with double-sided sticky carbon tape on a sample holder and kept in the preparation chamber overnight under vacuum to remove the adsorbed water from the dentine tubules. The full survey spectra were collected in a range of 0–1300 eV at pass energy, dwell time, and step size of 150 eV, 50 ms, and 1 eV, respectively.

### Mechanical properties

The ASTM D882 Test Method was followed for the measurement of the tensile strength (TS) and elongation at break (EAB) of the synthesized membranes. The specimens were equilibrated at a relative humidity of 50% in a desiccator. The measurements were performed on a Texture Analyzer (TA.XT plus, Stable Micro Systems, UK) using a 50-N load cell equipped with tensile grips (A/TG model) at 25 °C. The cross-head speed was 100 mm/min with a grip separation of 30 mm. Both TS and EAB were measured on ten random samples, and results are reported as the mean ± standard deviation.

### Barrier properties

The water vapor transmission rate (WVTR) of the membranes was measured using the gravimetric cup method as described by Hoque et al. [15] with a slight modification. For the WVTR measurement, deionized water (10 ml) was filled in a glass vial, its rim sealed with a circular film specimen (20 mm diameter), and kept in an incubator at 25.0 ± 1.5 ^◦^C and 45.0 ± 2.0% relative humidity. The water loss through the films was recorded by measuring the weight of each vial for 12 h at an interval of 60 min. The water loss recorded each hour was plotted against time, and the slope (g/h) of the line was determined. The slope (g/h) of the line divided by the film area (m^2^) gives the WVTR expressed as g /h.m^2^. Three measurements were performed for each membrane, and the data was reported as the mean ± standard deviation.

### Swelling analysis

The neutralized specimens were dried in an oven, and then immersed in 3 mL of PBS at 37 °C in an incubator. At regular intervals (0.25, 0.50, 1, 24, 28, and 72 h), the excess water was removed by dabbing the membranes on clean tissue papers, and the specimens were weighed. The percentage swelling ratio (Q) is calculated as follows:

$$\% Q=\frac{({W}_{w}-{W}_{d})}{{W}_{d}}\times 100$$

Where, W_w_ is the wet weight and W_d_ is the dry weight of the membranes.

### Degradation studies

Specimen dimensions used for swelling analysis and degradation studies were conducted in-vitro using 5 mg/mL of Egg Hen Lysozyme Solution (Sigma Aldrich, Waltham, MA, USA). The specimens were placed in a 24-well plate and immersed in 2 mL/well degradation media. The media was changed every 2 to 3 days, and pH was monitored at each time point. Degradation time points were taken on days 4, 7, 14, and 21. Degradation media were retrieved for conducting UV-vis spectroscopy. At each time point, specimens were washed twice with distilled water for 15 min and then dried in an incubator for 24 h before noting down the dry weight. They were also analyzed chemically using FTIR spectroscopy, and images of the specimens were taken using a DSLR camera. The weight loss percentage was calculated using the following formula:

$$W \% ={W}_{0}{\hbox{-}}\!{\hbox{-}}{W}_{1}/{W}_{0}$$

Where W_0_ is the weight noted before degradation, W_1_ is the weight noted after degradation analysis, and W% is the weight loss percentage.

### Drug release profile

Drug release studies were performed using semi-quantitative assessment of the release of Chlorhexidine digluconate in phosphate buffer solution (PBS) following the method described by Silva et al. [11]. The neat CH, CCG, 3511, and 3322 membranes were submerged in Falcon tubes filled with 15 mL of PBS solution. A sample of 1 mL was retrieved from this solution after the passage of pre-determined time points, and fresh PBS was replaced to maintain the total volume of 15 mL. The absorbance of each specimen was measured at 255 nm using a UV vis spectrophotometer (Eppendorf, BioSpectrometer^®^, Hamburg, Germany). Data was analyzed using a calibration curve that was prepared using the 5-parameter logistics curve fit as reported previously by Qasim et al. [16]. GraphPad Prism was used for data analysis (version 8.0).

### Microbial strains and culture conditions

Bacterial strains *Streptococcus mutans* CCUG 11877, *Aggregatibacter actinomycetemcomitans SA269, and Staphylococcus aureus CCUG* 43507 were cultured on brucella blood agar containing 5% sheep blood and incubated in 5% CO_2_ for 2-3 days. *Porphyromonas gingivalis* ATCC 33277 was grown on the same media for 3 days under similar conditions except an anaerobic atmosphere. All cultures were examined under a stereomicroscope to verify their colony morphology prior to use in actual experiments to ensure that there was no contamination.

### Bacterial biofilm culture

The bacterial strains (Section 2.10) were adjusted to an optical density (OD_600_) of 1. About 100 μl of the stock suspension was added to each well except for the control. Following the incubation period, the membranes were extracted from the wells using sterile forceps and transferred to a new set of sterile 24-well plates. To verify the absence of contamination during the experiment, a small sample was taken from the culture supernatant from each well to avoid contamination.

For the biofilm inhibition assay, the bacterial cells attached to the membranes and culture wells were collected in sterile microcentrifuge tubes. The tubes were washed twice by centrifugation at 5000 ×g for 5 min. After discarding the supernatant, the pellets were resuspended in sterile PBS. The suspensions were 10-fold serially diluted up to 10^−6^, plated on brucella blood agar, and incubated for 2-3 days as above.

For determining the total bacterial attachment to the membranes, membranes were washed three times with sterile PBS to remove unattached bacterial cells and any free DNA released by the microorganisms into the culture supernatants. Subsequently, the membrane discs were transferred to sterile microcentrifuge tubes containing 150 μl of nuclease-free water and vigorously vortexed for a min at the highest setting. The resulting microbial cell suspensions were then centrifuged at 10,000 × g for 5 min. After discarding the supernatant, the pellet containing the microbial cells was subjected to DNA purification. The centrifugation step ensured that only DNA from intact microbial cells was included in the quantification, excluding any extracellular DNA released by the microorganisms.

### DNA purification

The DNA obtained from both the reference microbial strains and membrane-detached microbial cells was purified using the DNeasy DNA Purification Kit from Qiagen GmbH (Hilden, Germany). For the enzymatic lysis step, a lysis buffer containing Tris-EDTA buffer (20 mM Tris, 2 mM EDTA) with 1.2% Triton X-100 and lysozyme was utilized (St. Louis, Missouri, USA). After purification, the DNA was eluted in nuclease-free water, and its concentration was determined using UV spectrophotometry (NanoDropTM 1000, Thermofisher, Waltham, Massachusetts, USA).

### Quantitative-polymerase chain reaction (qPCR)

For the qPCR analysis of the bacterial cells attached to the membranes, previously validated species-specific primers targeting the 16 S rRNA gene were employed [17–20] (Table 1). The qPCR reaction mixture consisted of 10 µl of SYBR Green master mix (Power SYBR Green Kit; Applied Biosystems, Waltham, Massachusetts, USA), 0.5 µl each of forward and reverse primers (0.2 µM), 7 µl of nuclease-free water, and 2 µl of DNA template.

**Table 1:** List of primers used for quantitative RT-PCR in this study

Species	Primer sequence 5’-3’	Target Gene	Annealing Temperature (^0^C)	Reference
*S. mutans*	F*:TCGCGAAAAAGATAAACAAACA R*: GCCCCTTCACAGTTGGTTAG	*htrA*	56	Chen et al. [17]
*A. actinomycetemcomitans*	F: TAGCCCTGGTGCCCGAAGC R:CATCGCTGGTTGGTTACCCTCTG	*16S rRNA*	68	Kim et al. [18]
*P. gingivalis*	F: AGGCAGCTTGCCATACTGCG R: ACTGTTAGCAACTACCGATGT	*16S rRNA*	52.2	Sakamoto et al. [19]
*S. aureus*	F: GCG ATT GAT GGT GAT ACG GTT R: AGC CAAGCC TTG ACG AAC TAA AGC	*nuc*	53	Zhang et al. [20]

*F* Forward, *R* Reverse

The qPCR amplification was performed on an ABI 7500 Fast RT-PCR machine (Applied Biosystems, Waltham, Massachusetts, USA) with the following temperature profile: an initial denaturation at 95 °C for 10 min, followed by 40 cycles of 15 sec at 95°C, 30 sec at 52–56 °C based on the specific primer pair, and 30 sec at 72 °C for elongation and fluorescent signal acquisition. The data were analyzed using the SDS 1.4.0 v software (Applied Biosystems, Waltham, Massachusetts, USA). To develop standard curves, the genomic DNA from the aforementioned species was serially diluted, and the cycle threshold (Ct) values were plotted against the deduced microbial cell content (cells/ml) for each species using the above software.

### Statistical analysis

All experiments were conducted in triplicates, and data analysis was performed using GraphPad Prism^®^ software (version 8.0.1; GraphPad Software Inc., San Diego, CA, USA). The results are presented as mean ± standard deviation. Data analysis was conducted using one-way analysis of variance (ANOVA). If a statistically significant difference was noted Student’s *t*-test for multiple comparison was used. For microbial counts, a non-parametric Mann-Whitney U-test was used to compare the differences between the groups. A *p*-value of < 0.05 was regarded as significant.

## Results

### Scanning electron microscopy

**Fig. 1:**
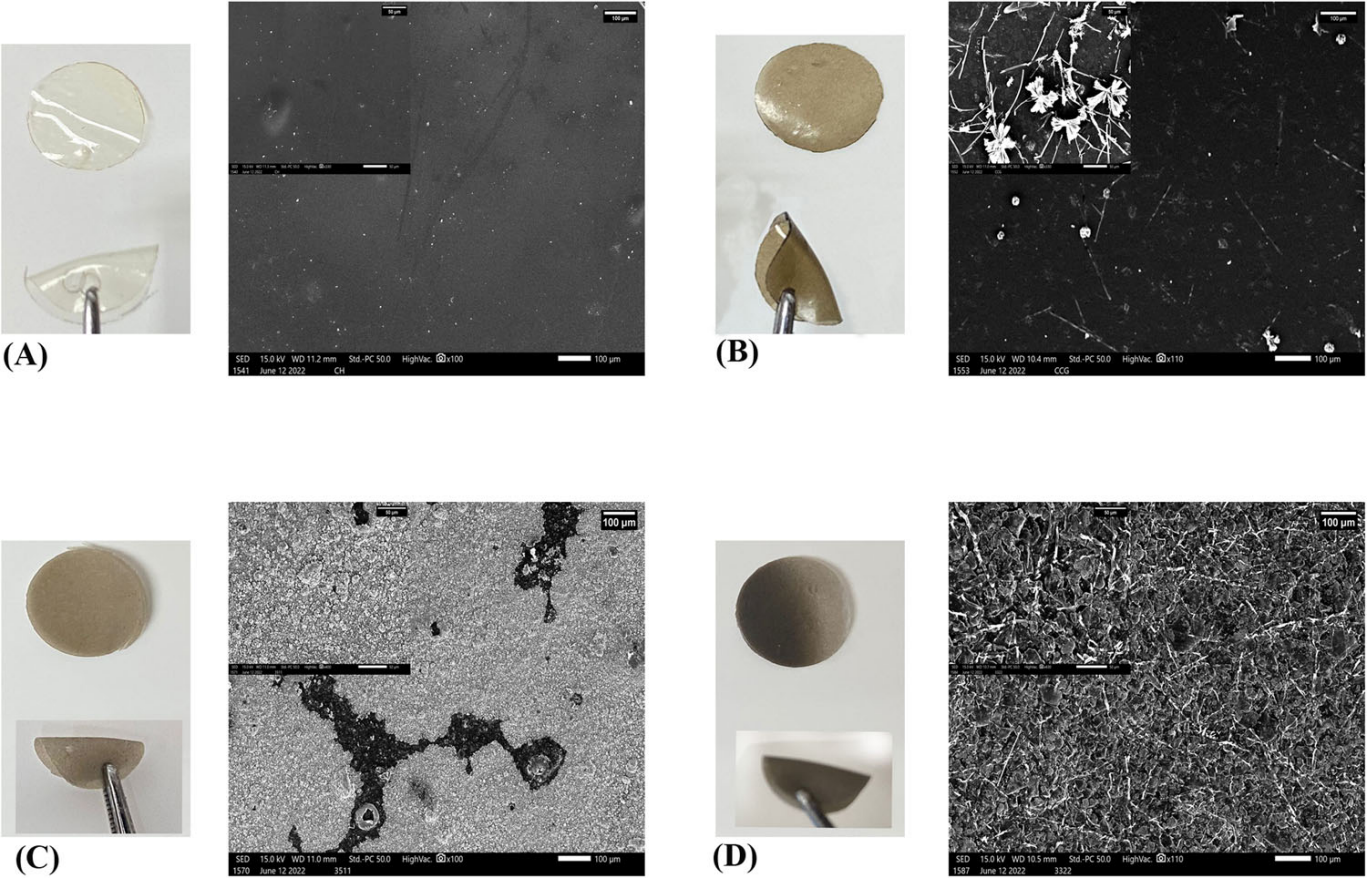
Optical images and scanning electron micrographs of (**A**) CH (**B**), CCG (**C**), 3511 and (**D**) 3322. SEM images are shown at a magnification of 100X and 330X inset images of the membranes are shown at higher magnification. Optical images showing the color change and handling characteristics of the membranes. Images are scaled at 100 µm and inset image at 50 µm

The optical microscopy observations of the membranes demonstrated that pure cellulose hydrogel (CH) exhibited transparency (Fig. 1A), while the incorporation of graphene oxide (GO) at specific concentrations led to varying degrees of darkening in the membranes (Fig. 1B–D). SEM results (Fig. 1A–D) revealed the alterations in the surface morphology upon compositional variations. The pristine CH membrane displayed a smooth and homogenous surface with a few small undissolved particles spread over the surface. The addition of GO and CHX in CCG induced the emergence of a distinct surface morphology, which was more evident at higher magnification. The addition of HA in different concentrations for 3511 and 3322 demonstrated a variation in morphological characteristics. HA particles were seen dominating the surface of 3511 in a tightly packed manner. The particle size of HA can also be observed in the higher inset image (Fig. 1C). The ultrastructure of 3322 showed a difference in morphology from CCG and 3511. Irregular HA particles can be observed, which are overlapped with thin fibers. These fibers are evenly distributed across the observed surface of the membrane. The transparency of CH and CCG can be observed clearly, whereas the addition of HA reduced this transparency.

### Fourier Transform Infrared Spectroscopy

**Fig. 2:**
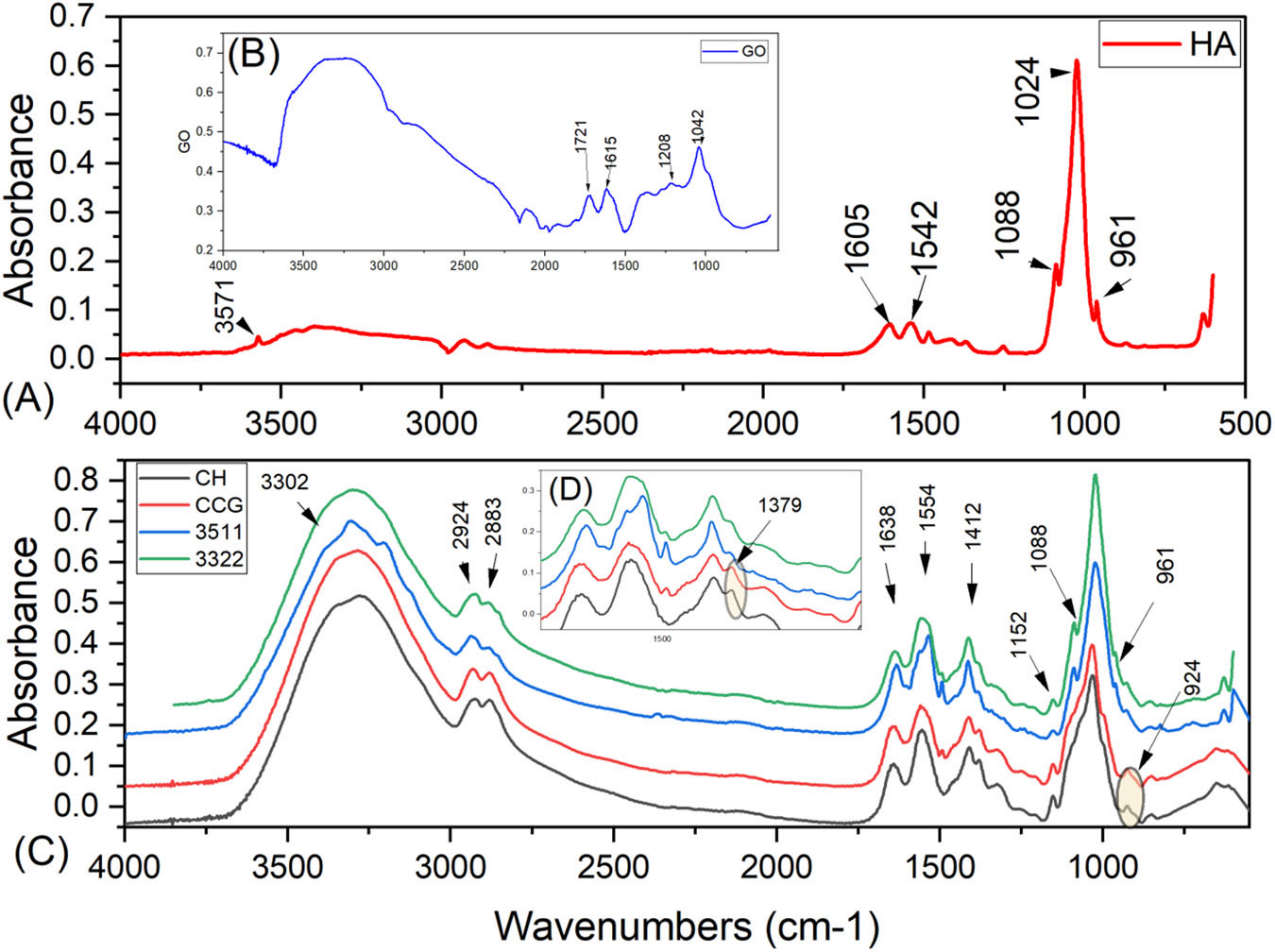
FTIR spectra (**A**) HA (**B**) GO (**C**) Prepared membranes neat CH, CCG, and composites 3511 and 3322. **D** The alterations in the fingerprint region. Peaks have been identified with their respective wavenumber numbers

FTIR spectral data of pristine GO, HA, and composite membranes are illustrated in Fig. 2. Pristine HA (Fig. 2A) reveals the typical peaks associated with phosphate vibrations (*v*_1_, at 961 cm^−1^, *v*_*3*_, at 1024–1088 cm^−1^ and *v*_*4*_ at 601–573 cm^−1^*)*. -OH peaks are also noted at 962.15 and 3500 cm^−1^ (Fig. 2A). FTIR of neat GO (Fig. 2B) shows a band at 3426 cm^−1^ assigned to hydroxyl group. Prominent peaks visible at 1721, 1615, and 1208 cm^−1^ are assigned to stretching vibrations of -OH, C = O, C = C, and C-OH, respectively. The peak at 1042 cm^−1^ is due to symmetric stretching vibrations of C-O-C. The GO spectra band associated with C-O (1042 cm^−1^) and C-OH at 1376 cm^−1^ was also noted. The broad band at 3395 and peak at 1615 cm^−1^ can be attributed to O-H stretching vibrations and aromatic C = C vibrations, respectively. The peak at 1721 cm^−1^ is associated with the vibration of C = O in carboxyl and carbonyl moieties. Neat CH (Fig. 2C) shows the glycosidic region (C-O-C), Amide I and Amide II peaks are quite evident. NH and -OH stretching vibrations near 3302 cm^−1^ can also be noted. The aliphatic C-H symmetric and asymmetric stretching vibration (2924 cm^−1^), C = N stretching vibration (1638 cm^−1^), and N-H stretching vibration are noted at 1554 cm^−1^, and C-O-C vibration is observed at 1026 cm^−1^. By comparing the neat and composite membranes, there was an obvious shift in the NH_2_ bands. Additionally, taking the peaks at 1638 and 1554 cm^−1^ as reference peaks, the primary amino group bending vibration band of CH, GO, HA, and CHX membranes showed alteration in the finger print region, which is attributed to the reaction between the epoxy group of GO and the primary amino group of CH (Fig. 2D).

### X-ray photoelectron spectroscopy

**Fig. 3:**
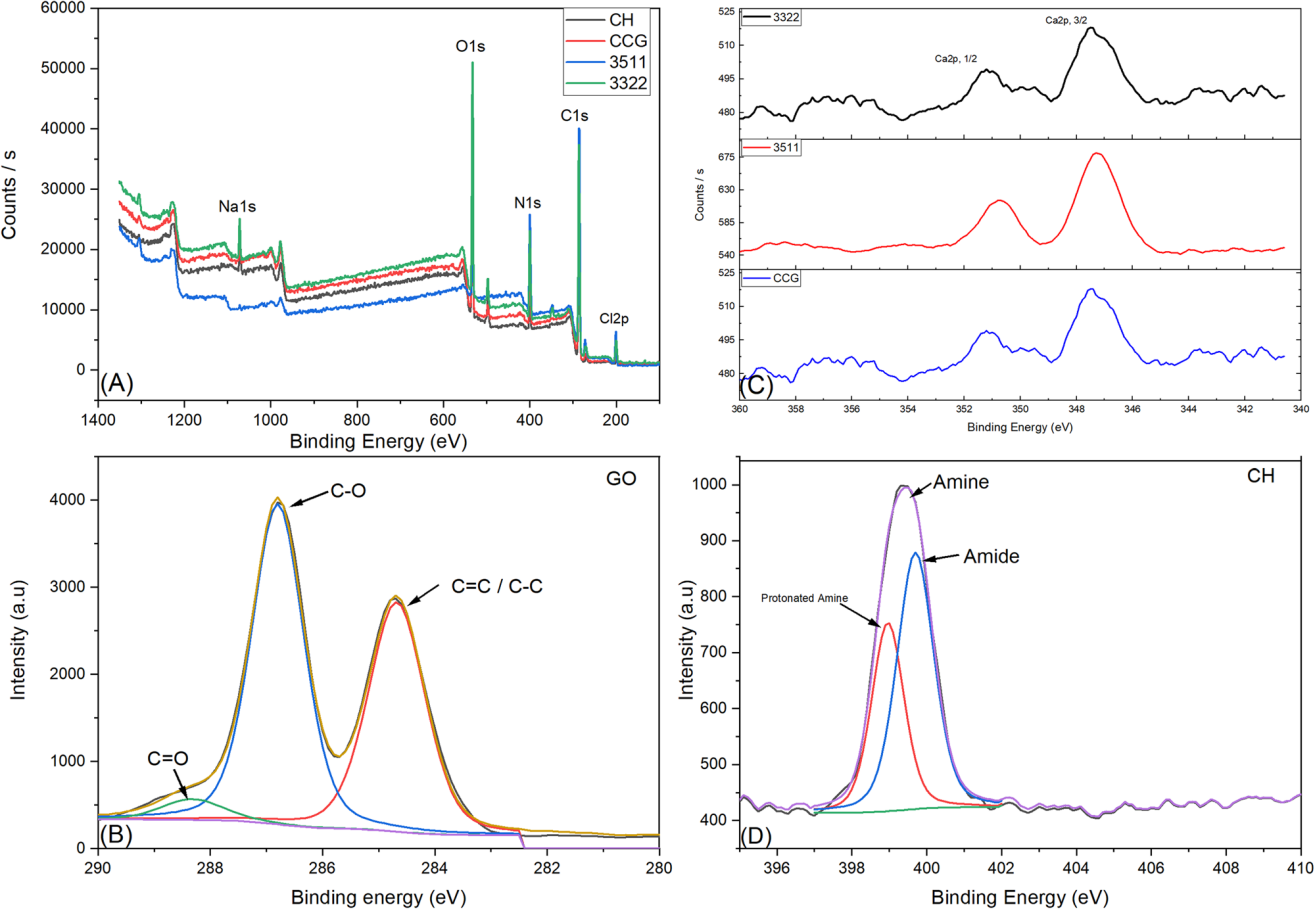
X ray photo electron spectroscopy data is shown in the form of Surveys of (**A**) CH, CCG, 3511 and 3322 (**B**) Carbon spectra of GO (**C**) Ca2p spectra of 3322, 3511 and CCG and (**D**) Nitrogen spectra of CH. More details of XPS spectra given in supplementary data

A high-resolution XPS spectral profile and quantitative XPS analysis were conducted on the developed membranes (Fig. 3; Table 2). Prominent peaks of Sodium (Na), Oxygen (O), Carbon (C), Nitrogen (N), and Chlorine (Cl) were detected in all membranes with different intensities, and the atomic percentage distributions were varied. Results demonstrated three different binding energies (284.68, 286.80, and 288.32 ev), which are GO assigned to carbon (C). The exhibited peaks for carbon atoms were due to hydroxyl, epoxy, carbonyl, and carboxyl groups, respectively (C = C/CC-C, C-O, and C = O). Fig. 3C revealed the double peaks of Ca2p _1/2_ and Ca2p _3/2_ with a separation of 3.57 eV. These are representative of Ca^2+^. The N in CH resulted in three different peaks that can be attributed to Amide (C-NHC = O), Amine (C-NH2), and protonated amine (C-NH^3+^) at 398.96 and 399.70. Results summarized in Table 2 show the variations in the atomic percentages or elements. The existence of Cl2p is unique to CHX. The high binding energy shoulder of 288.32 eV arises from imide carbon, which is also due to the CHX moiety. A detailed spectral profile of C, N, and O for 3511 and 3322 is reported as supplementary data.

**Table 2:** Summary of the atomic percentages of C 1 s, O 1 s, N 1 s, Ca 2p, Na, and P peak N1s, N1s A and N1s B refers to amine, amide and protonated amine

	CH	CCG	3511	3322
C1s	33.4	27.5	23.8	21.3
C1s A	6.3	3.8	5.0	4.6
C1s B	22.8	27.0	5.7	25.1
C1s C	6.3	8.2	4.1	8.9
O1s	0.5	4.9	1.2	5.2
O1s A	4.2	10.9	1.8	5.0
O1s B	9.0	7.8	1.9	13.9
N1s	10.2	2.8	2.0	1.4
N1s A	1.6	4.5	3.7	4.0
N1s B	-	-	2.5	5.0
Cl2p	-	1.6	2.4	2.8
Ca2p, ^³/²^	-	0.13	0.2	0.4
Ca2p, ^½^	-	-	0.1	0.2
P2p	-	0.1	-	0.4
Na1s	2.15	0.81	-	1.9

Where C 1s, C1s A, C1s B and C 1 s C refers to C-OH, C-O, C = O and C (O) O groups respectively

### Swelling ratio

**Fig. 4:**
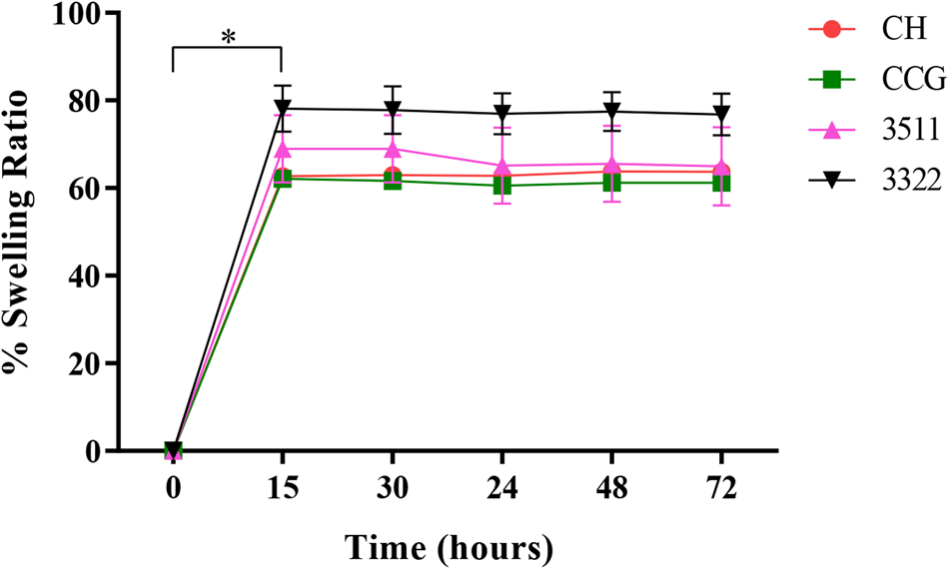
Water uptake analysis conducted by Swelling ratio (%) for a period of 72 h on CH, CCG, 3351 and 3322. Values showed are mean ± SD, where (*n* = 3). * denotes statistically significant difference

Results from the swelling analysis are represented in Fig. 4. Data shows that the specimens attain their maximum expansion after 15 min of soaking in the swelling medium and remain constant until the 72-hr experimental period. The highest swelling was noted by 3322 (80%) when compared with 3511, CCG, and CH alone (up to 60%) over the tested period. There was a significant difference in the swelling ratio at 0 and 15 min (*p* < 0.001). No significant difference was observed was observed in between the groups at 15, 30, 48 and 72 – hr.

### Mechanical and barrier properties

The tensile strength (TS) and strain% at the peak strength (SS) of neat CH membranes were 27.46 MPa and 4.03, respectively, which increased to 31.15 MPa and 8.74% with the addition of CHX and GO (Table 3). The TS values were further increased to 32.06 and 33.72 MPa with the incorporation of HA in the blend with two selected blend compositions (CHCG 3511 and 3320); however, the SS values did not follow the trend with the change in composition (15.64 and 9.08%). The elongation at break (EAB) of the neat CH improved marginally with the incorporation of GO nanoparticles and showed a further improvement in a quaternary blend containing 50% HA; however, it dropped with decreasing HA in the formulation of CHCG 3322. The improvement in the mechanical properties occurred because of the reinforcement of nanoparticles that dispersed well with increasing surface area in the CG matrix and improved the tensile properties. A decrease in the tensile properties in the formulation CHCG 3322 could be attributed to a lowering of the total contribution of nanoparticles ((HA + GO = 50 against 60 in other formulations).

**Table 3:** Results from the tensile tests showing the Tensile strength (MPa), Strain %, Elongation at break (EAB) % and water vapor transmission rate (Barrier g/h.m^2^)

	Tensile strength (MPa)	Strain% at the peak strength	EAB (%)	WVTR (g/h.m²)
CH	27.46 ± 4.67^a^	4.03 ± 0.70^a^	11.08 ± 2.89^a^	0.28 ± 0.01^ab^
CCG	31.15 ± 5.16^b^	8.74 ± 0.92^b^	11.89 ± 3.32^a^	0.23 ± 0.01^a^
3511	32.06 ± 5.41^b^	15.64 ± 1.24^c^	18.19 ± 4.60^b^	0.31 ± 0.01^b^
3322	33.72 ± 6.34^b^	9.08 ± 0.75^b^	9.78 ± 3.24^a^	0.30 ± 0.00^ab^

Values shown are mean ± SD

^*^Values in the same column with different alphabetic scripts indicate significant difference among samples at *p* < 0.05

A comparison of the barrier properties of neat chitosan and blended nanocomposite membranes demonstrated that the addition of 25% GO in the CH matrix improved the barrier property by decreasing the WVTR from 0.28 to 0.23 g/h.m². However, the WVTR values increased for both quaternary blends to 0.30 and 0.31 g/h.m², suggesting a decrease in water barrier property. Overall, nanocomposite films showed an improvement in barrier properties; however, the reverse trend could be attributed to nanocomposite adhesion, which might generate some cavities at the interface, resulting in more free volume and reducing the water barrier properties.

### Degradation analysis

**Fig. 5:**
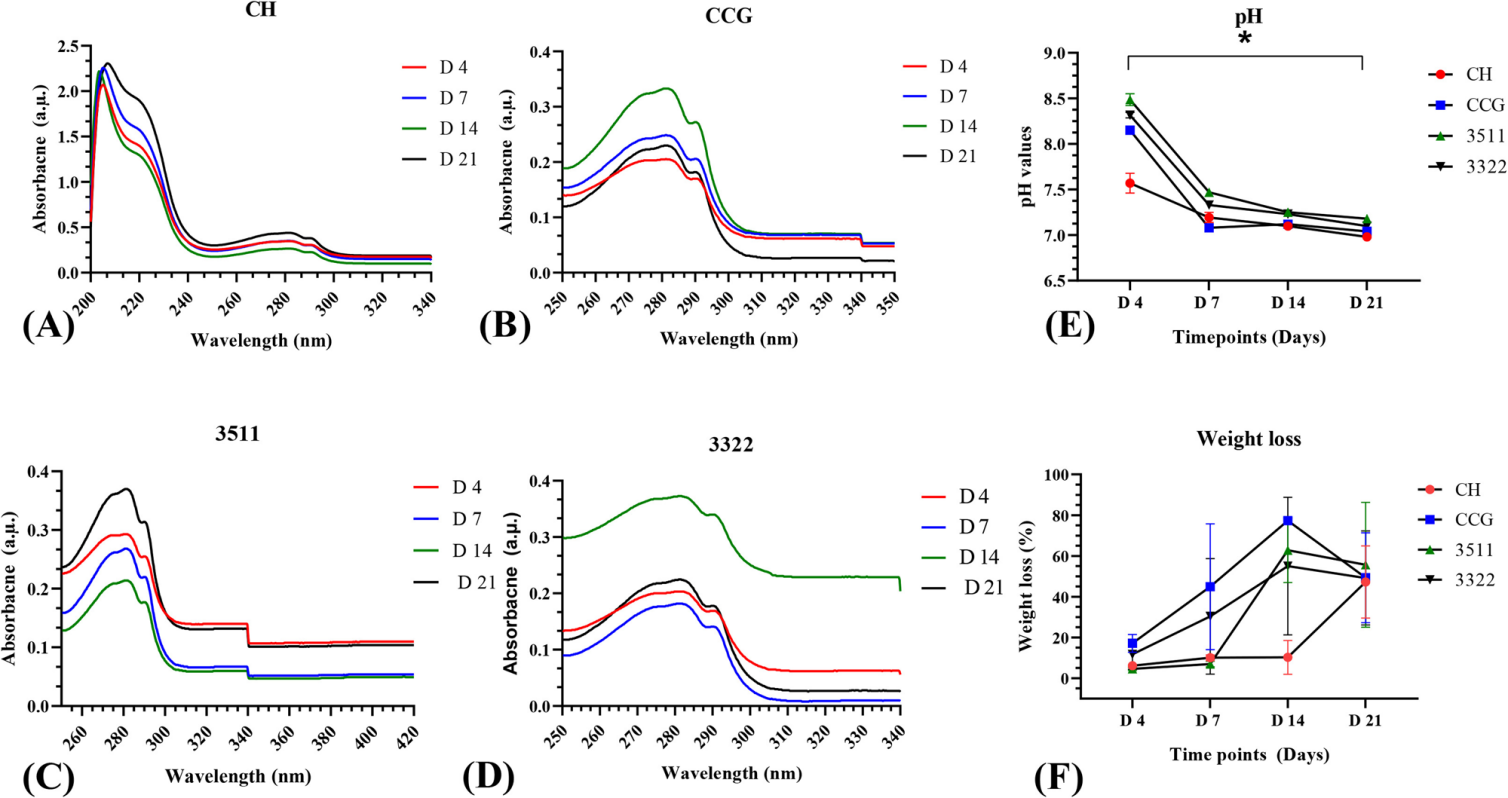
Degradation of the chitosan and composite membranes 3511, 3322 and CHC analyzed by (**A**)–(**D**) UV-vis spectroscopy at Day 4 (red), 7 (blue), 14 (green) and 21 (black) respectively (**E**) pH value change with time (**F**) Weight loss (%) analysis conducted over a period of 21 days. Specimens were analyzed on the Day, 4, 7, 14 and 21. Values of the pH and weight loss show mean ± SD

Results from the degradation study conducted by analyzing weight loss percentage (%), UV-vis spectroscopy, and pH value change are shown in Fig. 5A–F. UV-vis spectral data of neat CH degradation exhibited the occurrence of two absorption bands at 230 and 280 nm (Fig. 5A) with increasing intensity at day 21. CCG (Fig. 5B) also showed a strong band at 280 nm with an increment in intensity from days 4 to 21. With respect to 3511 and 3322, only the band at 280 nm was visible at different intensities (Fig. 5C, D). The pH values were monitored over a period of 21 days (Fig. 5E). On day 4, the values were observed to be 8.5 for CCG, 3511, and 3322, while for CH alone they were 7.5. The values dropped from day 4 to day 7, and at day 21, they attained a plateau from 7 to 7.5 (Fig. 5E). The weight loss (%) profiles of CH and composite membranes immersed in lysozyme solution for a period of 21 days are shown in Fig. 5F. CCG shows a relatively faster degradation from 20% on day 4 to almost 80% on day 14. Whereas, on day 21 weight loss (%) declined to 40%. A comparatively similar pattern was also noted to 3511 and 3322 after 14 days. CH alone was stable until day 14 and showed a weight loss of 40% on day 21.

### Drug release profile

**Fig. 6:**
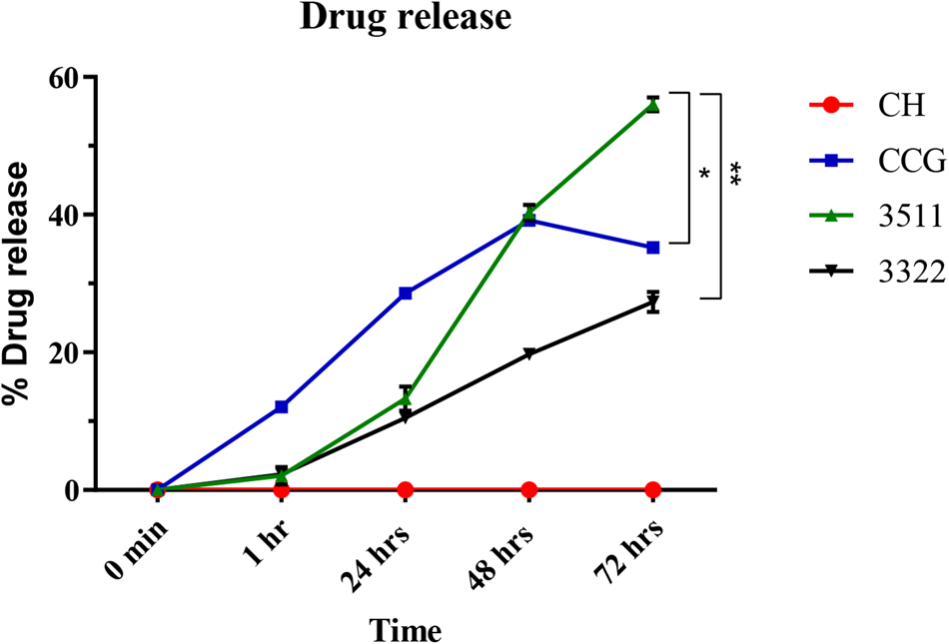
Cumulative release of chlorhexidine digluconate from CH and composite membranes over a period of 72 h at 37 °C. Results are expressed as percentage release compared to the entrapped amount and means with their respective SD (*n* = 3). Statistically significance is denoted by, * = *p* 0.01, ** = *p* < 0.0001

The drug release profile demonstrated significant differences in release profiles for CCG, 3511, and 3322 (Fig. 6). A gradual increment in the percentage of drug release over time was observed. The highest release profile is recorded for 3511, followed by CCG and 3322. 3322 showed the least drug release, with up to 25% release after 72 hr. No release was observed from CH. Further, a drop in the release profile was observed for CCG after 48 hr.

### Antibacterial analysis

#### Bacterial biofilm growth inhibition in the presence of the restorative materials

**Fig. 7:**
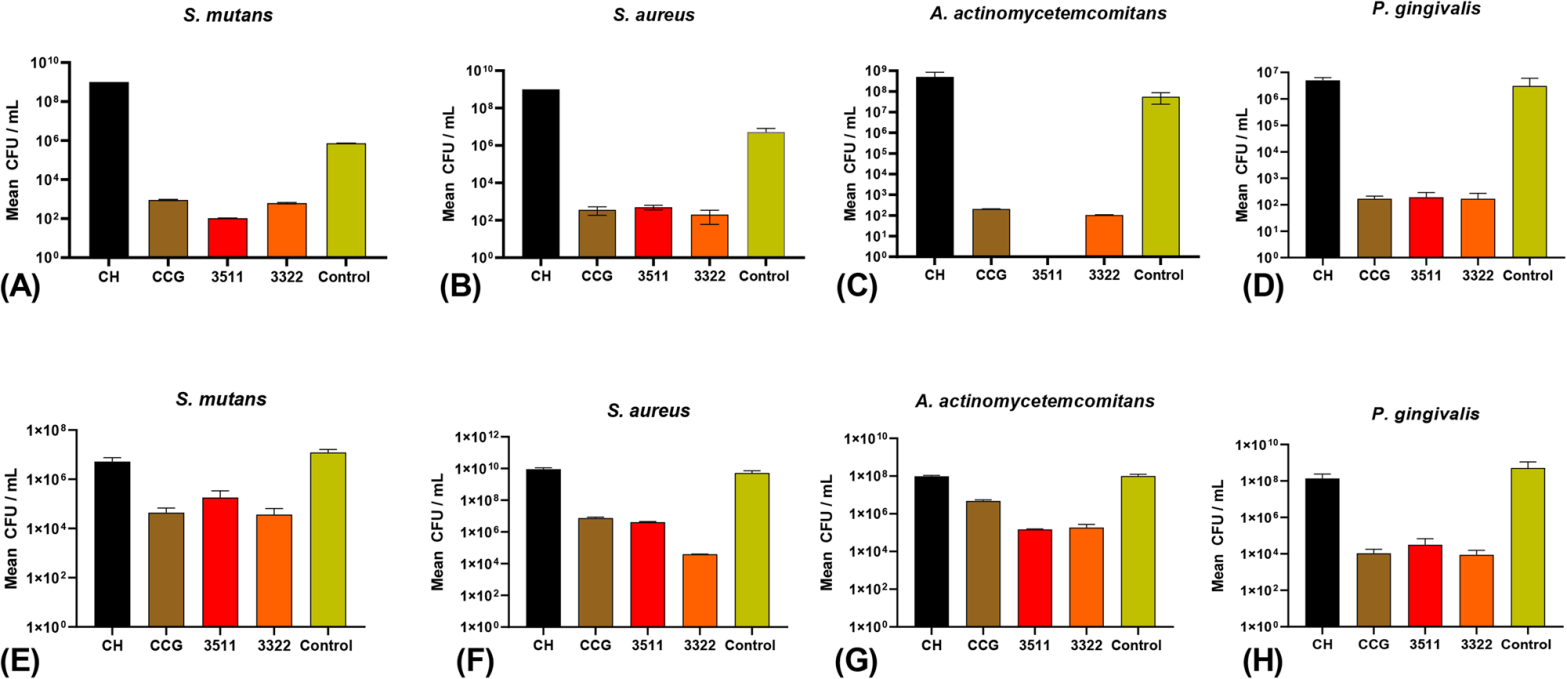
Antibacterial activity (**A**–**D**) and Quantitative RT-PCR results (**E**–**H**) of different membranes, CH, CCG, 3511 and 3322 on S. mutans, S. aureus, A. actinomycetemcomitans and P. gingivalis. Results represent the mean ± standard deviation of CFUs

Three different formulations of membranes were examined for their anti-biofilm potential. All three combinations of CH, CCG, 3511, and 3322, demonstrated significant biofilm growth inhibition against *S. mutans, P. gingivalis, A. actinomycetemcomitans*, and *S. aureus* (*P* < 0.05), when compared to CH individually or to the control specimen. The biofilm growth inhibition in the presence of CH was significantly higher for S*. mutans, A. actinomycetemcomitans*, and *S. aureus compared* to the control (*p* < 0.05). The 3511 showed complete inhibition against *A. actinomycetemcomitans*. (Fig. 7A–D).

#### Attachment of bacteria to the restorative membranes

In order to determine the extent of bacterial attachment to the membranes, we quantified all bacterial cells, live and dead, bound to the membranes. Quantitative RT-PCR analysis revealed that for all four bacterial species studied, the quantities of attached cells were significantly lower in CCG, 3322, and 3511 (*p* < 0.05), compared to CH or the control. Significant biofilm growth inhibition was observed with CH compared to the control (Fig. 7E–H).

## Discussion

Upon comprehensive literature review, it became apparent that a multifaceted biomimetic composition encompassing diverse nano biomolecules synergized with a pharmacologically established agent suitable for such defects could potentiate an amplified response concerning guided bone regeneration (GBR) whilst serving as a surface layer in a FGM. This amalgamation would ensure full disintegration within a predefined timeframe, thereby facilitating uninterrupted regeneration of the intricate cellular hierarchical microstructure. [21]. Periodontal lesions that have been treated with GTR have reported a favorable outcome in the past (75 - 88%), whereas without the use of such biomaterials, the success rate is reduced significantly (33–64.3%) [9]. Nevertheless, bacterial infection and colonization of membranes, followed by the spread of infection in the treated tissues, may trigger partial or complete failure of tissue regeneration. Overall findings revealed a strong chemical interactions of the molecules used for membrane fabrication and showed that the use of graphene oxide in low concentrations can be an effective strategy to counteract the bacterial load in GBR procedures.

Despite the apparent simplicity of the solution casting technique employed in polymer composites, it’s imperative to recognize that the incorporation of excessively rigid molecules into the polymers can exert an influence on the overall membrane thickness. During the casting of solutions, the polymer solution establishes a close and direct interface with the surface of the plastic petri dish [22]. As the solvent evaporates, the overall film shrinks due to solvent loss; this was also observed in the current study. Consequently, glycerol was introduced as a plasticizer to counteract this effect. The interaction between CH, GO, and HA has been studied in the past using different characterization techniques [23–25]. Optical images gave an indication of the flexibility and resilient nature of these membranes. Such properties suggest that the membrane can be conveniently adapted to the defect site even when used to combine with other membrane for the purpose of a surface layer in FGM. The images of CCG and 3322 showed lamellar crystals, which have also been reported previously [26]. Darkening of the membranes was due to the apparent addition of GO. The lower transparency of CCG could be due to the non-covalent physical adsorption of CH on both sides of the GO sheets [26]. HA has a proven record of triggering bone formation in in-*vivo* conditions [10, 11, 23, 27, 28]. Hence, it was added in different ratios to the membranes synthesized in this study. The inherent high specific surface area of GO aids in a higher loading level of HA particles. This is beneficial for forming an effective network inside the composite membranes. The benefit of having a dense layer is that it acts as a physical barrier, which can be beneficial when used in GTR or as a surface layer in FGM. In order to act as an occlusive barrier by preventing the connective tissue from invading into the defect side. Moreover, it is also preferred for guided bone regeneration since it permits osteoblast adhesion and blood clot stabilization [29]. The authors speculate that in light of the experimental outcomes, membranes denoted as 3511 and 3322 exhibit promising characteristics due to the presence of different bioactive molecules.

FTIR spectral data of pristine CH and composite formulations demonstrated that peaks and bands pertaining to CH were dominant. Some relevant peaks of phosphate vibrations (960 cm^−1^) of HA [2] were noted in the spectral data 3511 and 3322. These findings are in accordance with studies conducted previously [30, 31]. Since GO is chemically functionalized and is composed of sp^3^-hybridized carbon and sp^2^-hybridized carbon, the bands corresponding to these were noted in the spectral data of composite membranes [32]. The FTIR spectra indicated shifting of the wavenumbers and alterations in the intensity of the peaks with respect to carboxyl functional groups such as C = O and C-OH. Also, the glycosidic segment (C-O-C) group displayed similar shifts and intensity variations compared to pristine CH. These alterations were indicative of the fact that there were inter and intra molecular interactions in between GO, HA, CH and CHX possibly due to hydrogen interaction [27, 33]. XPS spectroscopy revealed that the C-C bond corresponds to the chemical bond formed by the carbon atom of the GO carbon skeleton *SP2*. The changes in the binding energies of 3511 and 3322 (supplementary data) of these carbon bonds are indicative of chemical bond formation between CH, HA, GO, and CHX. It can be speculated that GO can bind with CH and CHX via simple non-covalent interactions. Since CH is a hydrophilic biopolymer bearing a single amino group, there are two hydroxyl groups in the repeating hexosaminide residue, with their chemical activity following the order amino > primary hydroxyl >secondary hydroxyl. Hence, when GO is added to CH and CHX solutions, the negatively charged functional groups on the surface of GO will likely react with polycationic CH and CHX through either electrostatic interaction or hydrogen bonding mechanisms. Following this reaction, the calcium in HA might be attracted to -NH_2_ of CH through coordination bonding or by -OH of CH *via* electrostatic interactions [26]. These observations have also been reported by Qian et al. [34]. The results implied that there were significant structural transformations between CH, GO, HA, and CHX.

The mechanical and barrier properties of membranes provide insights into the clinical handling of the membranes during surgery and their stability on the defected site [35]. A continuous increase in the tensile properties with the loading concentrations of GO is consistent with the reported data in the literature [34, 36]. It has been reported that the mechanical properties of nanocomposites have a significant effect at low concentrations of GO-based fillers [26]. It could occur because of the homogenous distribution of GO in the CH, HA, and CHX matrix, which improved the stress transfer from the CH matrix to GO when a mechanical load was applied in tensile conditions [37]. Furthermore, this reinforcement may be due to the intrinsic mechanical properties of GO and good interfacial adhesion with the CH matrix (covalent bonding). It could be inferred that the values found were sufficient for the membranes to act as a barrier and could aid in structural support [29]. Similar results were also reported in other studies [37]. The swelling ratio of the membranes revealed that all membranes were hydrophilic, and 3322 had considerably higher water uptake. It could be due to the abundance of oxygen-containing functional groups. In particular, the ionization of carboxyl as well as the protonation of amino are believed to be able to improve the surface water’s wettability [34]. The observed performance of 3511 marked by better mechanical and barrier properties compared to CH, CCG and 3322 indicates the potential for enhanced efficacy and reliability of this formulation, making it a strong contender to translations use as GTR or as a surface layer in FGM.

Biodegradation of resorbable membranes in a timely manner is a complex phenomenon. The UV-vis spectral data showed two peaks that were indicative of hydrolytic scission of −1–4 glycosidic bonds of CH. These bands can also occur due to the -σ* (nonbonding to antibonding) transition of carbonyl or carboxyl groups and are indicative of bond breakage [38]. All biochemical reactions occurring in *vivo* have a direct and indirect correlation with pH. A drop in the values was noted from Day 4 to Day 7. This could be indicative of the attack of lysozymes on the amorphous zones since they retain more acidic species and are susceptible to degradation. Furthermore, this behavior can be attributed to the release of NH_2_ groups at the C-2 position as a result of bond cleavage in the lysozyme solution. Some of these ions release from CHX and GO complexes with OH ions, thereby increasing or decreasing the pH as the degradation proceeds [38]. A faster weight loss profile was observed for CCG, followed by 3322, 3511, and CH. Rapid degradation of scaffolds within the first two weeks of analysis has been reported earlier as well [28]. With respect to CCG membranes, the presence of CHX might accelerate degradation by disrupting the enzymatic process. Since a rapid early weight loss was noted in CCG this could be due to the burst release of CHX. After the passage of 14 days the chemical stability of CH and GO sustained this degradation process. Which was observed by the decline in the weight loss profile due to the strong molecular interactions in between the hydroxyl and amine groups of CH and the oxygenated functional groups on GO [30]. Overall, these membranes should functional for atleast 4 – 6 weeks to trigger successful regeneration of the periodontal system [4]. Although, the membranes studied in the current investigation demonstrated initial high strength (27 – 33 MPa), they completely lost their ultrastructural integrity within 4 weeks of incubation time in in-vitro conditions. Therefore, a crosslinking agent to strengthen the existing formulation could aid in prolonging the degradation rate in future [16].

CH alone and its combination with CHX have been readily used as drug delivery agents [39]. Moreover, CH-based systems have enhanced antibacterial activity when combined with CHX [39]. Drug release data revealed that there was a sustained release of CHX from CCG, followed by 3511 and 3322. This sustained release could be attributed to CHX being chemically bonded to GO and CH. Since CHX has a cationic charge, it bonds with CH and negatively charged GO in CCG membranes without hindering HA particles. The addition of HA to the composite membranes slowed the release. The discrepancies observed in the current release pattern could also be attributed to the concentrations of the drug added to each membrane. 3511, having the lowest drug, showed weaker interactions with CH, GO, and HA; hence, almost 60% were released after 72 h.

The antimicrobial potential of CH and CHX-based biomaterials has been well established in the existing literature [40]. GO alone and in composite formulations with other nanomaterials, due to its inherent properties, has also been exploited to exhibit strict antimicrobial activity [40]. However, the exact mechanism by which GO triggers the antimicrobial response is still unclear. Literature has suggested that the effects of GO are based on physical and chemical modes of action. Physical damages are most common and are triggered by direct contact of the sharp edge of graphene with bacterial membranes. Chemical modes are based on oxidative stress generated by charge transfer and reactive oxygen species [41]. Although all tested membranes showed significant growth inhibition of bacteria and qPCR revealed lower levels of attached cells, The variations in the observed results could be due to the variations in the concentrations of GO, CHX, and CH in each membrane. The highest concentration of GO was present in CCG, followed by 3322 and 3511, respectively. Furthermore, the synergistic effect of HA, GO and CHX as observed in 3511 and 3322 contributed to antimicrobial properties. These properties combined with the physical, chemical and mechanical characteristics suggest that 3511 could be more promising choice for use in GTR or as a surface layer in FGM. Although it may seem that GO has an antibacterial effect on bacteria and biofilms, it has been reported that less than 50 g/mL of GO serves as a nutrient medium and acts as a biofilm to enhance bacterial growth [6]. It can be speculated that the combination of biomolecules used in the current formulations may have a multispecies effect on bacteria.

## Conclusion

In this study, simple solvent casting was used to prepare chitosan and composite membranes with graphene oxide, chlorhexidine digluconate, and hydroxyapatite. The mechanical properties and handling characteristics in dry and wet conditions revealed that they can be used with ease at a chairside procedure in a clinical setup. The chemical analysis suggests that a strong interaction can be achieved based on the availability of functional groups. The unique physical and chemical characteristics of these nanocomposites work together in a synergistic manner to reveal a highly efficient antimicrobial membrane. Based on degradation studies, drug delivery profiles, and antimicrobial analysis, it can be speculated that 3511 can be used as a surface layer in a functionally graded membrane for guided bone regeneration.
